# From tradition to evidence-based medicine: A two-decade bibliometric analysis of acupuncture efficacy, neural mechanisms, and therapeutic integration (2005–2024)

**DOI:** 10.1097/MD.0000000000051353

**Published:** 2026-11-06

**Authors:** Jingxuan Peng, Hong Xu, Jiawei Yang, Junhao Liang, Yuting Lv, Xiaohong Yao, Ting Yin

**Affiliations:** a Affiliated Guangdong Hospital of Integrated Traditional Chinese and Western Medicine of Guangzhou University of Chinese Medicine, Foshan, Guangdong, China; b Shuguang Hospital Affiliated to Shanghai University of Traditional Chinese Medicine, Shanghai, China.

**Keywords:** acupuncture, bibliometric analysis, data visualization, mechanism, pain

## Abstract

**Background:**

Acupuncture has gained widespread recognition as a therapeutic intervention for various conditions, yet a comprehensive bibliometric analysis of global research trends in this field remains lacking.

**Objectives:**

This study aimed to analyze the characteristics, research hotspots, and emerging frontiers of acupuncture studies worldwide from 2005 to 2024.

**Methods:**

A systematic search was conducted in the Web of Science Core Collection, focusing on highly cited articles. Data extraction included publication year, citation counts, research themes, author affiliations, and international collaborations. Statistical and network analyses were performed using VOSviewer and SPSS.

**Results:**

The top 100 most-cited articles were published between 2014 and 2021. The United States was the leading contributor (51 articles), followed by China (29 articles) and Germany (23 articles). Citation counts for these articles ranged from 127 to 757. The highest number of articles was published between 2005 and 2010. The average annual citation rate showed an increasing trend, while a negative correlation was observed between total citations and annual citation counts. Klaus Linde from the Technical University of Munich was the most prolific author. The *Cochrane Database of Systematic Reviews* contributed the most papers (8) to the top 100 list. Research hotspots included acupuncture mechanisms (30%), particularly neural pathways for pain relief, and clinical applications (58%), such as chronic pain and mental health management.

**Conclusion:**

This study provides a bibliometric analysis of the 100 most-cited articles in acupuncture research, highlighting key trends, contributors, and research focuses. The findings offer valuable insights for future studies.

## 1. Introduction

Acupuncture, a cornerstone of traditional Chinese medicine, involves inserting fine needles into specific acupoints to stimulate healing.^[1,2]^ Recognized globally since the 18th century as a complementary therapy, it is widely used for neuropathic pain, chronic disorders, and complex conditions.^[3–5]^ Traditional Chinese medicine theory posits that health relies on the harmonious flow of “*qi*” (vital energy), which acupuncture aims to rebalance.^[1]^ Modern research indicates that acupuncture activates peripheral nerves, triggering spinal and supraspinal reflexes to modulate the immune and nervous systems.^[6]^ For instance, it enhances endorphin release and suppresses pro-inflammatory cytokines, thereby alleviating pain and inflammation.^[7,8]^ These mechanisms align with the body’s innate regulatory processes.^[9]^ Furthermore, contemporary research on acupuncture does not supersede the classical concept of “qi” but rather provides biological validation and mechanistic translation of this traditional framework, as exemplified by mechanistic studies and large-scale clinical trials.^[10,11]^ Compared to pharmaceuticals like nonsteroidal anti-inflammatory drugs – which carry risks of gastric ulcers, dyspepsia, and gastrointestinal bleeding – acupuncture’s adverse effects (e.g., minor bruising or transient dizziness) are infrequent and generally mild, as evidenced by clinical safety data.^[12]^ This favorable profile makes acupuncture a nonpharmacological treatment option with high efficacy and low risk.

Bibliometric analysis employs mathematical and statistical methods to evaluate published articles, revealing disciplinary trends and advancements in specific fields.^[13]^ Citation quantification serves as a key metric, indirectly reflecting the quality, influence, and academic impact of research outputs.^[14]^ Therefore, a comprehensive analysis of highly cited articles can illuminate future directions for a discipline.^[15]^ Bibliometric analysis has been widely applied to acupuncture therapy research, covering diverse areas such as pain,^[16]^ facial paralysis,^[17]^ stroke,^[18]^ or sleep disorders.^[19]^ However, most existing studies focus on individual subfields; a comprehensive bibliometric assessment of acupuncture therapy remains lacking. To address this gap, our study analyzes the top 100 most-cited articles over the past 2 decades, aiming to outline current research status and emerging trends in the field.

## 2. Materials and methods

### 2.1. Searching strategy

This study performed a systematic literature search using the Web of Science Core Collection, with search terms derived from the PubMed keyword list (TS=(“Acupuncture” OR “Electroacupuncture” OR “ Pharmacopuncture”)). The search covered publications from January 1, 2005, to December 31, 2024, without language, document type, or study design restrictions. Results were sorted by descending citation frequency, with more recent publications receiving priority when citation counts were equal, accounting for their shorter time available for citation accumulation. The selection focused on studies where acupuncture was the primary intervention, excluding those where acupuncture appeared among multiple treatments, addressed unrelated subjects, or were conference abstracts/case reports. Two independent researchers screened records to identify the top 100 most-cited articles, resolving discrepancies through discussion. Ethical approval was unnecessary as the study involved no human/animal experimentation or primary data collection.

### 2.2. Data extraction

The 100 most-cited acupuncture articles were documented in a spreadsheet, recording titles, total citations (TC), annual citation rates (average citations per year = citations/[2023 − publication year + 1]), journal names, and publication years. Articles were classified as original research, comments, letters, or editorials through post-search verification. Two investigators (HX and YL) analyzed abstracts and keywords to determine research themes. Journal Impact Factors came from the 2023 Journal Citation Reports.

### 2.3. Statistical analysis and bibliometric methods

Qualitative data are expressed in percentage frequency. Quantitative data are represented as the median (first quartile [Q1], third quartile [Q3]) and subjected to normality testing using the Kolmogorov–Smirnov test. Group comparisons used Mann–Whitney *U* tests, while bivariate relationships employed Spearman correlations (significance threshold *P* < .05). Statistical processing utilized SPSS. Charts were generated using software such as Excel, VOSviewer, Scimago Graphica, etc. In network visualization maps, different nodes represent different elements, such as country/region, author, or keyword. The size of a node reflects the number of outputs, references, or occurrences, and the color represents the different clusters or average years of occurrence of these elements. Lines represent the relationships between elements, while connections between nodes represent co-occurrence associations.

## 3. Results

A total of 29,159 articles were obtained from the Web of Science Core Collection database. The top 100 most-cited articles were ranked in descending order by TC count (Table S1, Supplemental Digital Content 1). Among the T100 articles on acupuncture, there were 58 original articles, 41 reviews, and 1 letter.

### 3.1. Year of publication

The publication years of the T100 articles span from 2005 to 2021. Figure 1 shows the annual number of publications during this period. Overall, the number of highly cited articles exhibited a fluctuating downward trend, with most high-impact publications concentrated between 2005 and 2010. The peak year was 2005 (n = 14), followed by 2007 (n = 13), after which the annual output declined (Fig. 1).

**Figure 1.**
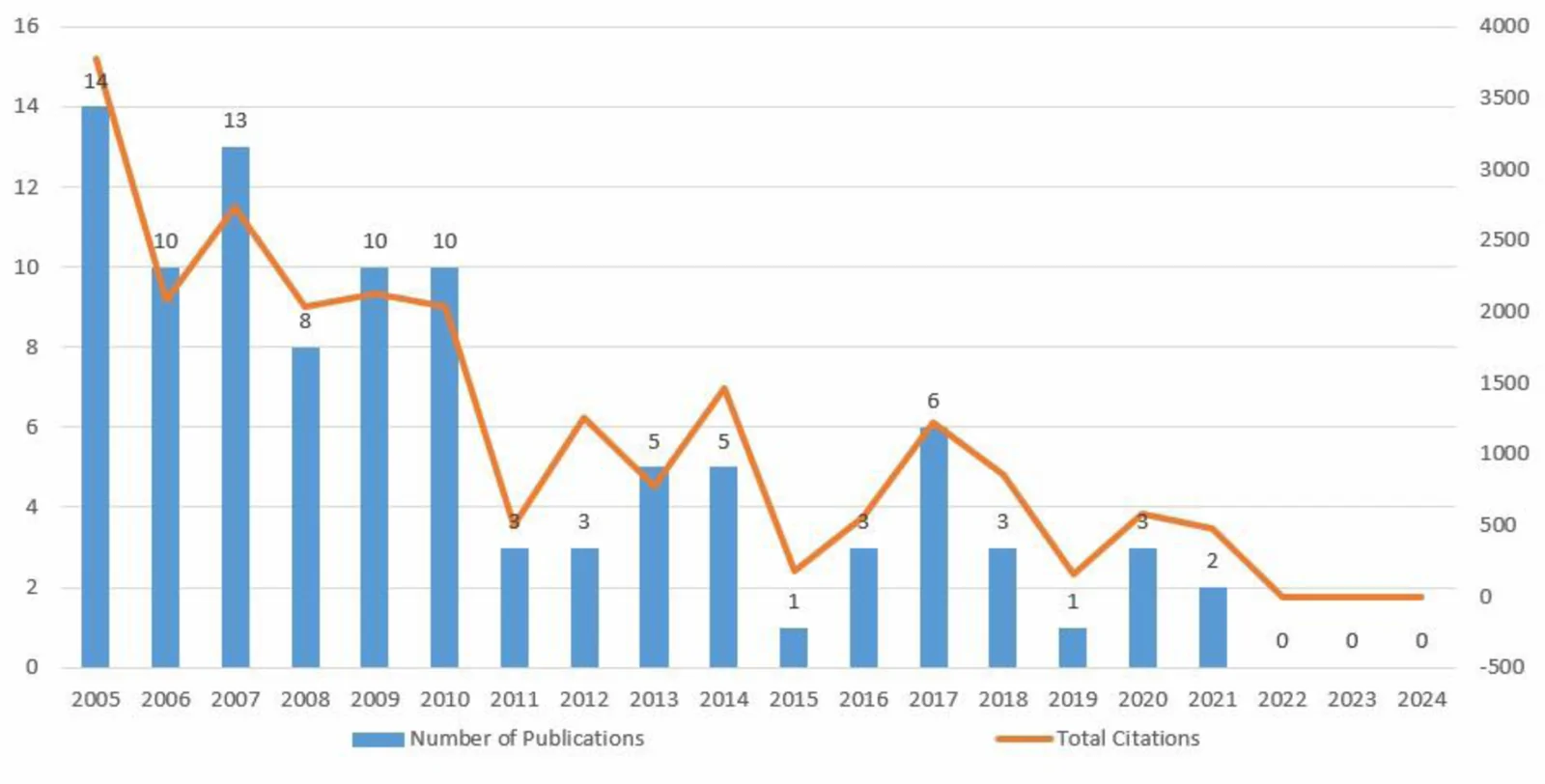
Number of top-cited publications from 2005 to 2024.

### 3.2. Citations

The 100 articles had citation counts ranging from 127 to 757 per article, with a total of 22,784 citations across all publications 186 (146, 264). The top 10 most-cited articles accounted for 23.3% of TC (n = 5313). The annual citation rate ranged from 6.45 to 87.00 with a median of 13.38 (9.68, 21.42).

Articles published between 2015 and 2024 had a significantly higher median annual citation rate 28.00 (21.44, 32.00) compared to other articles 11.73 (9.14, 15.12; *P* < .001), but no difference in TC (198 [154, 232] vs. 180 [145.5, 275.0]; *P* = .878). Correlation analysis revealed no significant association between TC and journal impact factor (IF; *r* = 0.122, *P* = .242) or publication year (*r* = −0.040, *P* = .703). Controlling for publication year did not alter the TC-IF relationship (*P* = .245), confirming year was not a confounder.

Figure 2 shows trends in total and annual citations in each 5-year interval. The average annual citations were constantly ascending. TC were negatively correlated with annual rates (*r* = −1.000). However, due to the limited sample size, the significance is insufficient, and the statistical power is low (Fig. 2).

**Figure 2.**
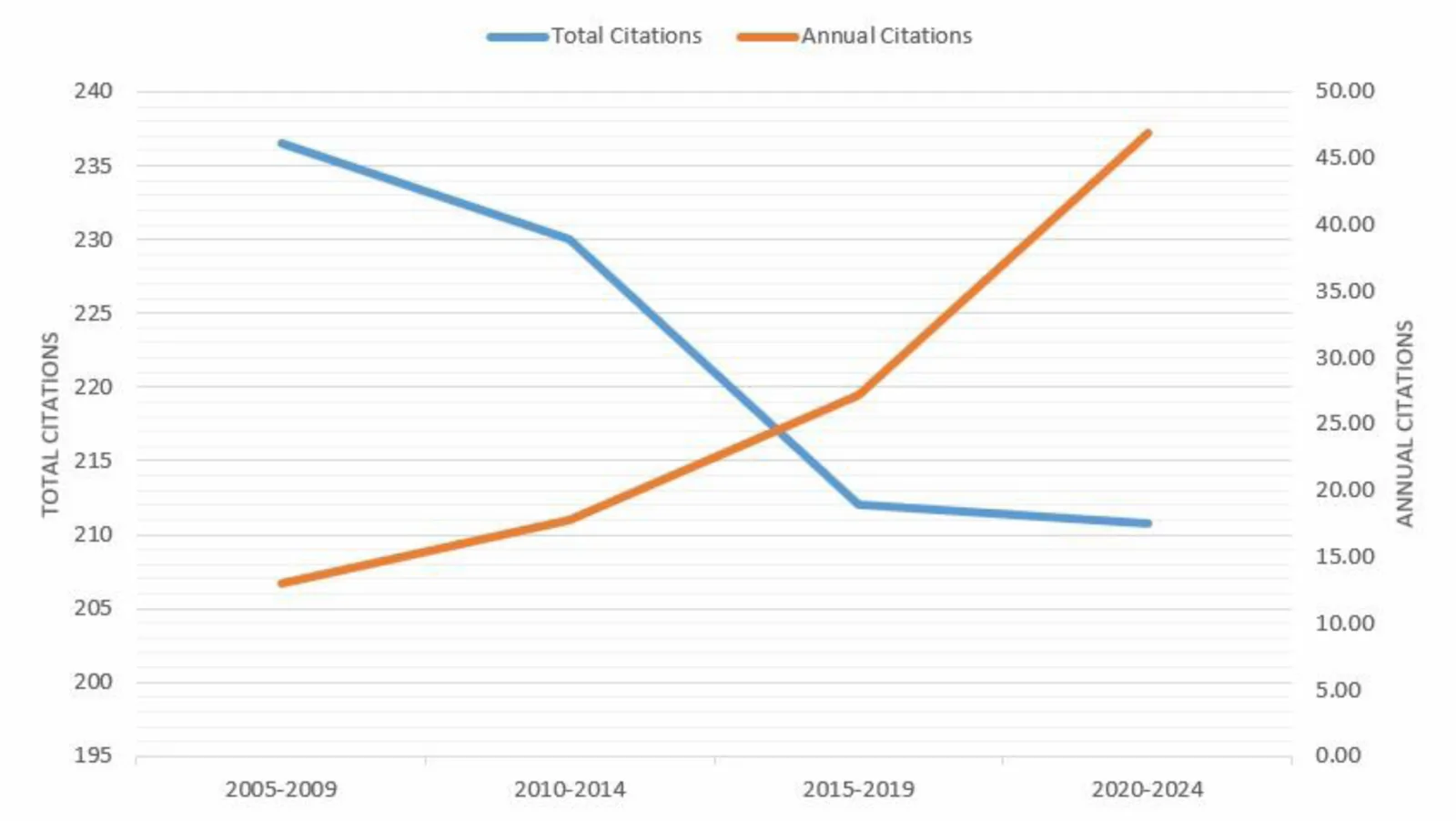
Citation analysis of the top 100 most-cited articles in each 5-year interval.

### 3.3. Article type and research focus

Among the 100 most-cited articles, 58 were original articles, 41 were reviews, and 1 was a letter. No significant difference was observed in the median TC counts between original articles 186 (142, 284.8) and reviews 191 (152.5, 234; *P* = .879). Similarly, the annual citation rates did not differ significantly between the 2 groups, with original articles 13.43 (8.75, 22.55) versus reviews 12.73 (10.50, 18.61; *P* = .670).

The 100 most-cited articles were categorized into different research focuses: treatment (58%), mechanism (30%), safety (2%), effectiveness (2%), and others (8%). Among the 58 treatment-related studies, 66% focused on pain management, with the most common research directions being lower back pain (24%), bone and joint pain (24%), and migraine (13%). The remaining 34% covered non-pain-related applications, such as cancer care, insomnia, depression, and stroke. Of the 30 mechanism studies, 40% investigated the regulatory effects of acupuncture on neural pathways, representing the primary research focus. In addition, 23% explored analgesic mechanisms, 10% analyzed anti-inflammatory mechanisms, and the remaining 27% examined other aspects, including mechanical effects, blood flow improvement, and intestinal microbiota regulation.

In these 100 articles, 31 were randomized controlled trials (RCTs). Notably, 27 of these 31 RCTs compared acupuncture with a placebo control. The acupuncture group showed positive outcomes in 22 trials and no significant effects in 5, whereas the placebo group had 9 effective and 18 ineffective trials. A chi-square test revealed that the effectiveness rate of the acupuncture group (81.4%) was significantly higher than that of the placebo group (33.3%), (*χ*^2^ = 12.80, *P* < .001), with a moderate-to-large effect size (Phi = 0.49). In addition, the relative risk (RR = 2.44, *P* < .05) indicated that acupuncture increased the likelihood of a positive outcome by 2.44-fold, and the odds ratio (OR = 8.77) further supported its superiority over placebo.

### 3.4. Contributions of countries

The analysis of 100 included articles identified authorship contributions spanning 17 countries/regions, with 9 countries/regions meeting the threshold of ≥5 articles. Author geographical distribution was visualized using Scimago Graphica (Fig. 3A), while international collaboration networks were constructed using VOSviewer (Fig. 3B).

**Figure 3.**
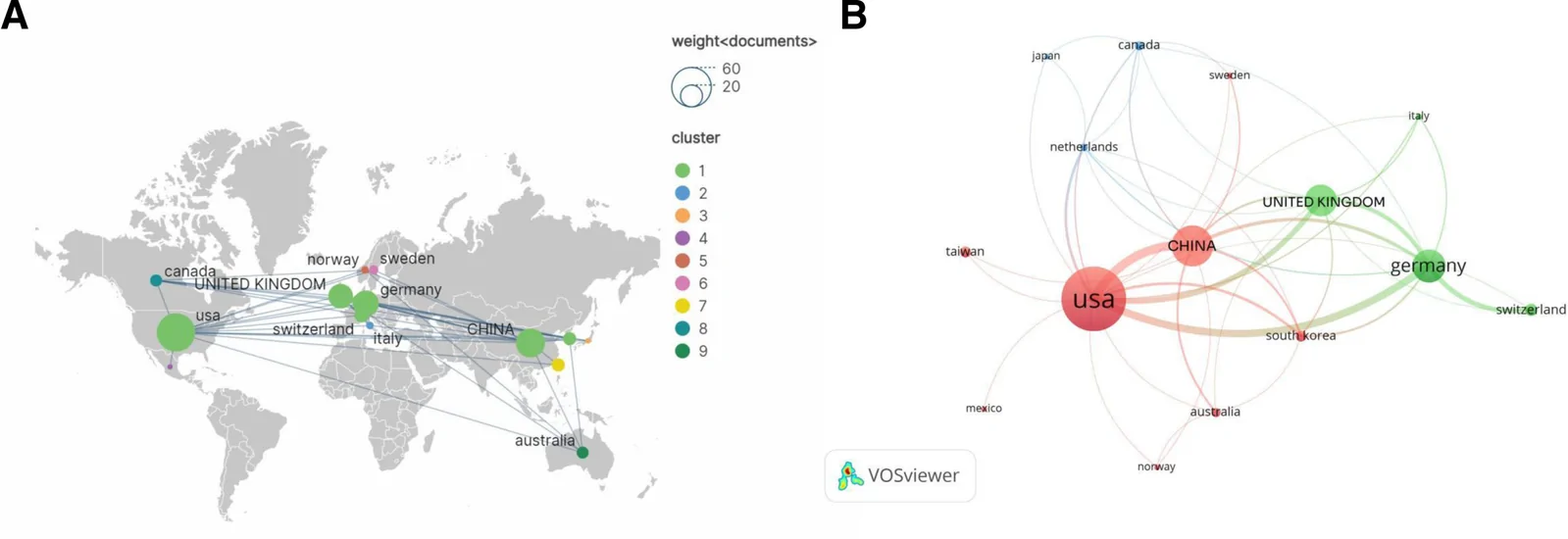
(A) Distribution maps of authors from different countries in the top 100 most-cited articles. (B) Map of the collaborative network between countries.

The USA emerged as the predominant contributor (n = 51), followed by China (n = 29), Germany (n = 23), and the UK (n = 21). Network analysis confirmed the USA’s leadership in both scholarly impact and collaborative intensity, with particularly strong cooperative ties to China, Germany, and the UK (Table S2, Supplemental Digital Content 2).

### 3.5. Contributions of institutions

A total of 56 academic institutions contributed to the 100 articles (Table S3, Supplemental Digital Content 3). Technical University of Munich (TUM) ranked first with 14 publications with a total of 4440 citations, followed by the University of Maryland (n = 13) and Massachusetts General Hospital (n = 12). Network analysis revealed TUM’s strongest collaborative partnerships with Charite University Medical Center, University Zurich Hospital, and the University of Munich (Fig. 4).

**Figure 4.**
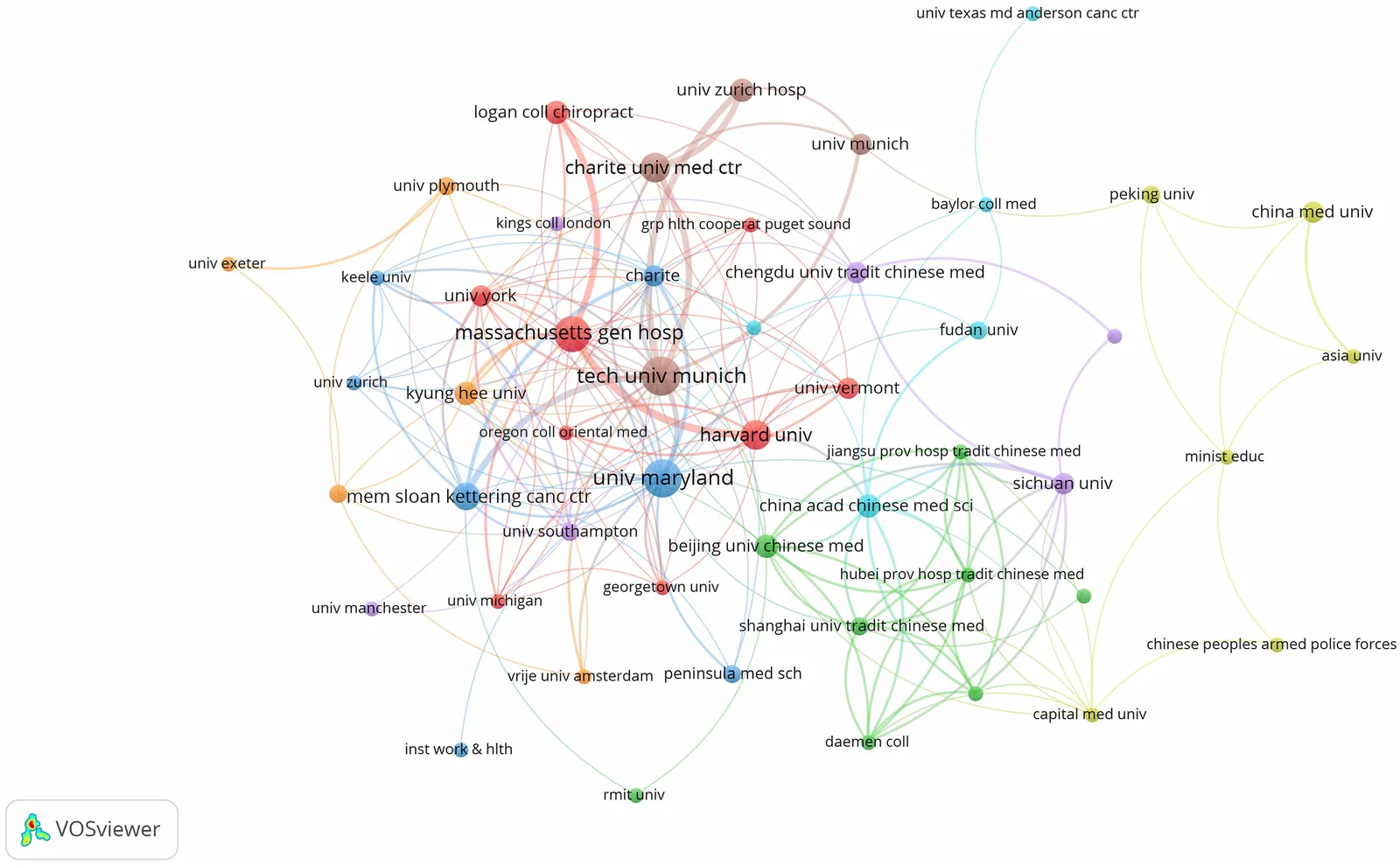
Map of the collaborative network between institutions.

### 3.6. Contributing of authors

Analysis of the 100 articles identified contributions from 66 distinct authors (Table S4, Supplemental Digital Content 4). Table 1 demonstrates that 8 authors made significant contributions by producing more than 5 highly referenced publications. The most productive author was Linde K from TUM (n = 14), followed by Witt CM (n = 10) and Brinkhaus B (n = 9) from the Berlin Institute of Health. Citation analysis revealed Linde K as the most-cited author (n = 4440), with network analysis identifying this researcher as having the strongest collaborative connections (TLS = 76). The author coauthorship network (Fig. 5) displays distinct research clusters through different colors, demonstrating established collaborative patterns among research teams (Table 1).

**Table 1:** Top 10 authors in the top 100 most-cited articles.

Author	TP	TC	CPP	TLS	Institution	Country
Linde K	14	4440	317.1	76	Technical University of Munich	Germany
Witt CM	10	3867	386.7	58	Berlin Institute of Health	Germany
Brinkhaus B	9	2912	323.6	38	Berlin Institute of Health	Germany
Berman BM	9	2155	239.4	20	Virginia Commonwealth University	USA
Napadow V	8	2068	258.5	27	Spaulding Rehabilitation Hospital	USA
Willich SN	7	2460	351.4	48	Berlin Institute of Health	Germany
Lao LX	7	1599	228.4	21	Virginia University of Integrative Medicine	China
Macpherson H	5	1678	335.6	21	University of York	UK
Melchart D	4	1503	375.8	37	Deggendorf Inst Technol	Germany
Streng A	4	1503	375.8	37	University of Wurzburg	Germany

CPP = citations per publication, TC = total citations, TLS = total link strength, TP = total publications.

**Figure 5.**
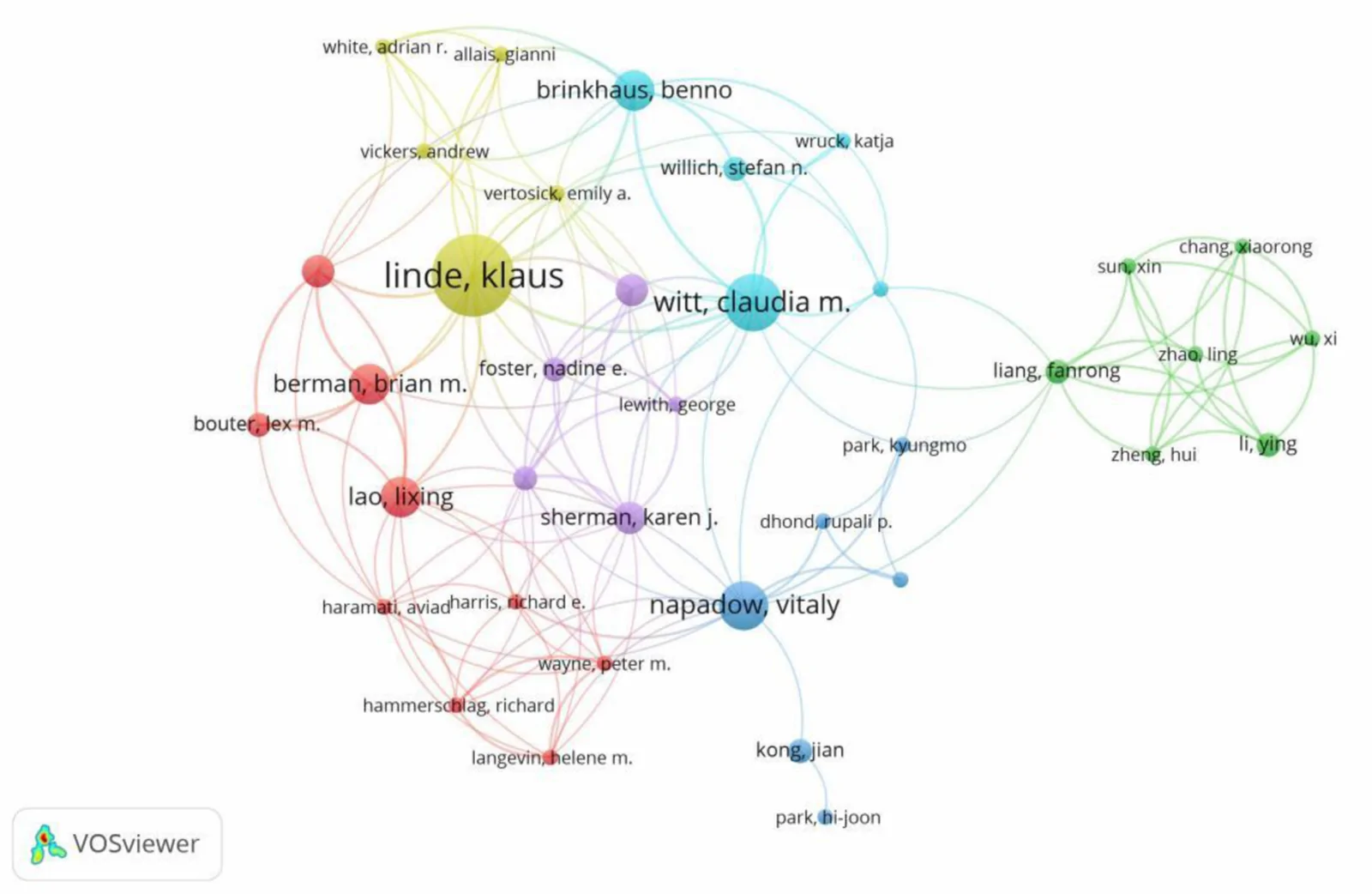
Map of the collaborative network between authors.

### 3.7. Distribution of journals

The 100 articles were published across 58 journals, with Table 2 listing the 10 most prolific journals by publication count. *Cochrane Database of Systematic Reviews* led with 8 articles, followed by *JAMA-Journal of the American Medical Association* and *BMJ-British Medical Journal* (n = 6 each). Archives of Internal Medicine emerged as the most-cited journal (n = 1817). Per Journal Citation Reports, 90% of these top journals were Q1-ranked, with *BMJ-British Medical Journal* having the highest IF (IF = 93.7; Table 2).

**Table 2:** Top 10 journals with the most publications.

Title	TP	TC	CPP	JCR	IF 2023
Cochrane Database of Systematic Reviews	8	1704	213	Q1	8.8
JAMA – Journal of the American Medical Association	6	1400	233.3	Q1	63.5
BMJ – British Medical Journal	6	1228	204.7	Q1	93.7
Annals of Internal Medicine	5	1089	217.8	Q1	19.6
Journal of Clinical Oncology	5	883	176.6	Q1	42.1
JAMA Internal Medicine (formerly Archives of Internal Medicine)	4	1817	454.25	Q1	23.3
Pain	4	1016	254	Q1	5.9
Neuroimage	3	832	277.3	Q1	4.7
Journal of Pain	3	768	256	Q1	4
PLoS One	3	653	217.7	Q1	2.9

CPP = citations per publication, IF = impact factor, JCR = Journal Citation Reports, TC = total citations, TP = total publications.

### 3.8. Recent articles

No articles published after 2021 were identified in our initial screening. To explore potential recent trends, we conducted a supplementary search for the most-cited articles published between 2022 and 2024, with the top 10 listed in Table S5, Supplemental Digital Content 5.

The citation counts ranged from 50 to 104, with a median of 70.5 (59, 91.5), and the annual citation rates ranged from 17.67 to 34.67, with a median of 25.16 (21.83, 33.12). The TC count of the top 100 articles was significantly higher than that of these articles (*P* < .001). In contrast, the annual citation rate of these articles was significantly higher (*P* = .001). This discrepancy indicates that while recent studies have lower cumulative citations, their higher annual citation rates demonstrate accelerated early-stage impact. This pattern may suggest emerging interest in these topics, although long-term citation trends remain to be observed.

Among the 10 most-cited 2022 to 2024 articles, 5 were reviews, 3 were original articles, and 2 were editorials. Mechanistic studies represented 50% of the articles (primarily on anti-inflammatory or immunomodulatory pathways), followed by therapeutic research (30%, covering clinical applications such as insomnia and anxiety) and evidence-based medicine research (20%, focusing on acupuncture trial design and evidence evaluation).

## 4. Discussion

### 4.1. Basic information

Bibliometric analysis provides a quantitative approach to examine publication characteristics.^[20]^ Within this framework, the top 100 most-cited articles are widely regarded as seminal works that represent influential research in a given discipline.^[21]^ Applying this methodology, the current study investigates research trends in acupuncture through an analysis of the most frequently cited publications in this specialized field.

The analyzed publications span from 2005 to 2021, with the period between 2005 and 2010 representing the most productive phase for influential works. The United States emerges as the leading contributor in both publication output and citation impact, establishing its predominant position in this research domain. China and Germany follow as primary collaborators in international research networks. In assessing research impact, while citation frequency may not serve as an absolute quality indicator, it effectively reflects academic recognition.^[22]^ Publications garnering over 400 citations are conventionally classified as landmark studies.^[23]^ Our analysis identifies ten such seminal papers, with research foci distributed as: pain management (n = 6), analgesic mechanisms (n = 3), and anti-inflammatory mechanisms (n = 1). This distribution highlights the predominant research directions in contemporary acupuncture studies. A noteworthy finding reveals that merely two of these highly cited papers represent basic research originating from Chinese scholars. This stands in contrast to the more balanced basic/clinical research contributions from international authors. This disparity persists despite China’s extensive historical experience and substantial clinical trial activity in acupuncture, suggesting potential gaps in research depth or global engagement of Chinese contributions.^[24]^

The research institutions are predominantly concentrated in North America and Europe, regions that typically benefit from well-established research infrastructures and funding mechanisms.^[25]^ Within this institutional landscape, 3 centers demonstrate particular research excellence: the TUM, specializing in clinical investigations of acupuncture analgesia and placebo effects^[26,27]^; The University of Maryland’s Complementary Medicine Research Center, a pioneer in evidence-based acupuncture research^[28,29]^; and Massachusetts General Hospital, which employs functional MRI technology to elucidate acupuncture’s effects on brain networks.^[30,31]^ The analysis of institutional productivity reveals 2 noteworthy patterns. First, among the top 10 most influential institutions, only 2 are Chinese-based, suggesting that Chinese research centers could benefit from enhanced publication strategies and deeper international collaborations to increase their global impact. Second, despite the United Kingdom ranking as the fourth most productive country in terms of total publications, no British institutions appear in the top 10. This discrepancy may reflect the relatively dispersed nature of acupuncture research across UK institutions, coupled with insufficient institutional coordination mechanisms.^[16]^ These findings underscore the importance of both international collaboration and domestic institutional partnerships in building research capacity and visibility.

Our author affiliation analysis reveals distinct collaborative patterns in acupuncture research, with particularly robust networks among European and American researchers. Professor Linde K stands out as the field’s most prolific contributor, with 14 seminal publications to his credit. This leading researcher has cultivated extensive collaborative networks across multiple teams, specializing in clinical studies and systematic evaluations of acupuncture efficacy. His influential 2010 meta-analysis demonstrated that using sham acupuncture significantly impacts clinical trial design and interpretation.^[32]^ Other prominent contributors include Witt CM (n = 10) and Brinkhaus B (n = 9), among other established researchers. The collaboration network analysis indicates that Chinese researchers exhibit significantly less international collaboration than Western scholars, maintaining predominantly domestic research networks – a pattern potentially attributable to both their greater publication output in Chinese journals and stronger emphasis on traditional theoretical approaches. These findings highlight the importance of strengthening institutional collaborations and fostering international partnerships to advance acupuncture research globally. The next frontier lies in global partnerships that synergize Eastern clinical expertise with advanced neuroimaging technologies, such as functional MRI and PET-based receptor imaging, alongside Western biostatistical rigor.^[30,31]^ Such collaborations would empirically substantiate traditional principles using cutting-edge tools, ensuring these therapies are preserved, validated, and optimized for future generations.

Our analysis indicates that the majority of the most highly cited articles are published in the *Cochrane Database of Systematic Reviews*, followed by *JAMA-Journal of the American Medical Association* and *BMJ-British Medical Journal*. This pattern may reflect both the exceptional quality of articles in these journals and researchers’ tendency to preferentially cite high-impact publications.^[33]^ Notably, while *JAMA Internal Medicine* ranks sixth among the top 10 journals by publication volume (with only 4 papers), it leads in both TC (TC = 1817) and citations per paper (CPP = 454.25), demonstrating its outstanding influence in this research field. This makes *JAMA Internal Medicine* particularly valuable for researchers to reference and emulate. Furthermore, most of these leading journals possess high IF, supporting the well-established correlation between journal impact factor and research quality.^[34]^

### 4.2. Research hotspots and frontiers

The top 100 most-cited articles cover a wide range of topics related to acupuncture. By analyzing keywords and article types, we identified current research hotspots and potential future directions in acupuncture therapy.

Among the 100 articles, 30 focus on the mechanisms of acupuncture, with the neural mechanisms of acupuncture analgesia being the primary research direction. The most representative study is the 2008 paper “Neural Mechanism of Acupuncture Analgesia” published in Progress in *Neurobiology*, which, with 757 citations, has become the most frequently cited research in this field. It systematically elucidates that the essence of acupuncture analgesia lies in the multilevel integration of nociceptive signals from pain regions and afferent impulses from acupoints within the central nervous system, offering a neurobiological translation of the classical “qi” framework.^[10]^ Subsequently, the 2010 study “Adenosine A1 Receptor-Mediated Local Analgesic Mechanism of Acupuncture” published in *Nature Neuroscience* further expanded the field by exploring the relationship between acupuncture and purinergic signaling, establishing molecular correlates of acupoint-specific effects.^[11]^

From a research perspective, current studies on acupuncture analgesia mechanisms primarily unfold at 2 levels: cellular and molecular level: multiple signaling molecules, including opioid peptides and glutamate, mediate acupuncture’s analgesic effects. Particularly, opioid peptides and their receptors (μ, δ, and κ subtypes) play pivotal roles in this process.^[35,36]^ Furthermore, electroacupuncture (EA) at different frequencies induces frequency-dependent opioid peptide release. For instance, 2 Hz EA primarily triggers enkephalin release in the spinal cord, while 100 Hz EA promotes dynorphin secretion, thereby differentially alleviating inflammatory and neuropathic pain.^[37]^ Brain functional level: fMRI studies reveal that acupuncture modulates neural activity across multiple brain regions, including the somatosensory cortex, limbic system, basal ganglia, brainstem, and cerebellum. Notably, low-frequency EA induces broader fMRI signal enhancement than manual acupuncture.^[30,31]^ However, the specific neural pathways and precise brain regions involved require further elucidation.^[38]^ These multilevel findings collectively provide a comprehensive understanding of acupuncture analgesia mechanisms and establish a robust scientific foundation for its clinical application. The high citation impact of these studies reflects both methodological rigor and empirical validation of an ancient observational science that anticipated multilevel regulatory mechanisms now being elucidated through neuroimaging and molecular pharmacology.

A systematic analysis of the 100 articles revealed that 58 (58%) primarily focused on therapeutic applications. Among the top 10 most-cited articles, 6 investigated the efficacy of acupuncture in pain management, including chronic pain, low back pain, migraines, and knee osteoarthritis. This distribution underscores that validating the analgesic effects of acupuncture remains a central research priority. A particularly influential 2012 systematic review (cited 725 times) provided robust evidence supporting acupuncture’s effectiveness in treating chronic pain conditions such as low back pain, neck pain, osteoarthritis, chronic headaches, and shoulder pain.^[39]^ Chronic pain management emerged as the predominant clinical focus, constituting a modern validation of the traditional principle of unblocking meridians to alleviate stasis.^[26,39]^

Of note, our analysis of the 31 RCTs within the top 100 corpus demonstrated an 81.4% effectiveness rate for acupuncture versus 33.3% for placebo controls (RR = 2.44, *P* < .05), indicating that acupuncture increases the likelihood of a positive outcome by 2.44-fold. This favorable efficacy profile is particularly meaningful when weighed against the safety risks associated with conventional pharmacotherapy. As noted in the Introduction, NSAIDs – commonly prescribed for pain relief – carry well-documented risks of gastric ulcers, dyspepsia, and gastrointestinal bleeding.^[12]^ In contrast, acupuncture is associated with infrequent and generally mild adverse events, such as minor bruising or transient dizziness, as supported by clinical safety data. Taken together, these findings position acupuncture as a safer nonpharmacological alternative to NSAIDs for pain management, offering robust therapeutic benefits without the systemic toxicity inherent in long-term drug use.

In addition to pain management, numerous studies have explored acupuncture’s role in treating anxiety, depression, and related comorbidities. Cancer and depression exhibit a bidirectional relationship that may mutually exacerbate disease progression.^[40]^ Among the top 100 articles, several high-impact reviews and RCTs have specifically examined acupuncture for depressive disorders. A 2018 Cochrane review and a 2010 systematic review with meta-analysis both provided evidence supporting the safety and efficacy of acupuncture as an alternative or adjunctive treatment for depression, highlighting its potential as a nonpharmacological intervention for mood disorders.^[41,42]^ Furthermore, one RCT within our corpus specifically evaluated acupuncture for posttraumatic stress disorder, demonstrating positive outcomes.^[43]^

Sleep disturbances constitute another domain where acupuncture has shown considerable promise. Our analysis identified a RCT on primary insomnia, which demonstrated the efficacy and safety of acupuncture treatment, with significant improvements in sleep quality and duration.^[44]^ Insomnia frequently coexists with chronic pain and mood disorders, and the presence of acupuncture-related RCTs on insomnia within the top 100 list reflects the growing recognition of acupuncture’s role in comprehensive sleep management.

Cancer-related fatigue represents a third major area of clinical expansion. Two RCTs in our corpus specifically addressed cancer-related fatigue in breast cancer patients, both demonstrating significant improvements with acupuncture treatment.^[45]^ In addition, a systematic review of acupuncture in cancer care synthesized evidence across multiple studies, supporting acupuncture’s role in alleviating fatigue, anxiety, and depressive symptoms in cancer populations.^[46]^ A 2020 meta-analysis on cancer pain further reinforced the value of acupuncture and acupressure in oncology supportive care.^[33]^ Research by Molassiotis et al demonstrated that acupuncture may have considerable potential in alleviating cancer-related fatigue.^[47]^ Evidence-based medicine also supports the safety and efficacy of acupuncture as an alternative treatment for depression, highlighting its promise as a nonpharmacological intervention for both pain and mood disorders.^[41]^ Collectively, these findings from high-quality RCTs and systematic reviews within our top 100 corpus substantiate acupuncture’s role as a holistic therapeutic modality that addresses the multifaceted nature of patient health – encompassing pain, mental health, sleep quality, and cancer-related symptoms – thereby positioning it as a valuable nonpharmacological strategy within integrated care models.^[41,45,46]^

However, some studies suggest that, in addition to the specific effects of acupuncture, other factors such as acupoint selection variability and psychological effects may act as confounding variables in assessing acupuncture’s therapeutic efficacy.^[48,49]^ Within the current evidence-based medical paradigm, high-quality clinical trials employing rigorous methodologies are essential to establish reliable scientific evidence for acupuncture’s efficacy. Future research should further address these confounding variables to strengthen clinical validity.

## 5. Conclusion

This study conducted a comprehensive bibliometric analysis of acupuncture research from 2005 to 2024 using the Web of Science database. Our analysis of the T100 articles revealed that the United States dominated. The evaluation of cited authors and institutions suggested that international collaboration should be strengthened to enhance the global impact of acupuncture research. The main research focus centered on investigating the mechanisms and therapeutic applications of acupuncture, particularly its molecular and neural mechanisms as well as analgesic effects. However, several limitations should be acknowledged. First, restricting the search to a single database (Web of Science) and excluding non-English publications may have resulted in the omission of relevant studies. Second, our article selection based on title and abstract screening might have led to underrepresentation of significant research. These limitations may affect the comprehensiveness of our findings.

In conclusion, this study identified and analyzed the bibliometric characteristics of the 100 most-cited articles in acupuncture therapy, providing valuable insights that may facilitate future research in this field.

## Author contributions

**Conceptualization:** Jingxuan Peng, Hong Xu, Jiawei Yang.

**Data curation:** Jingxuan Peng, Xiaohong Yao.

**Project administration:** Jingxuan Peng.

**Methodology:** Hong Xu, Junhao Liang.

**Software:** Hong Xu, Jiawei Yang.

**Supervision:** Ting Yin.

**Writing – original draft:** Jingxuan Peng, Hong Xu, Jiawei Yang, Junhao Liang, Yuting Lv.

**Writing – review & editing:** Yuting Lv, Ting Yin.










