# Supplementary material for: From tradition to evidence-based medicine: A two-decade bibliometric analysis of acupuncture efficacy, neural mechanisms, and therapeutic integration (2005–2024)
Source: Medicine (Baltimore). 2026 Nov 6;105(45):e51353. doi: 10.1097/MD.0000000000051353 (PMC13747620; doi:10.1097/MD.0000000000051353)
Supplement: Supplementary file 1 [file medi-105-e51353-s001.pdf]

Title	TC	ACY	Journal	Year	IF	Number	Type	Research
Neural mechanism underlying acupuncture analgesia	757	44.53	PROGRESS IN NEUROBIOLOGY	2008	6.7	1	article	mechanism
Acupuncture for Chronic Pain Individual Patient Data Meta-analysis	725	55.77	ARCHIVES OF INTERNAL MEDICINE	2012	17.3 (2014)	2	review	treatment
Adenosine A1 receptors mediate local anti-nociceptive effects of acupuncture	588	39.20	NATURE NEUROSCIENCE	2010	21.3	3	article	mechanism
Mechanisms of Acupuncture-Electroacupuncture on Persistent Pain	531	48.27	ANESTHESIOLOGY	2014	9.3	4	article	mechanism
Acupuncture for Chronic Pain: Update of an Individual Patient Data Meta-Analysis	481	68.71	JOURNAL OF PAIN	2018	4	5	review	treatment
German acupuncture trials (GERAC) for chronic low back pain -: Randomized, multicenter, blinded, parallel-group trial with 3 groups	468	26.00	ARCHIVES OF INTERNAL MEDICINE	2007	17.3 (2014)	6	article	treatment
Acupuncture for patients with migraine -: A randomized controlled trial	454	22.70	JAMA-JOURNAL OF THE AMERICAN MEDICAL ASSOCIATION	2005	63.5	7	article	treatment
Acupuncture in patients with osteoarthritis of the knee: a randomised trial	450	22.50	LANCET	2005	98.4	8	article	treatment
Dopamine mediates vagal modulation of the immune system by electroacupuncture	443	40.27	NATURE MEDICINE	2014	58.7	9	article	mechanism
The impact of patient expectations on outcomes in four randomized controlled trials of acupuncture in patients with chronic pain	416	23.11	PAIN	2007	5.9	10	review	other

The integrated response of the human cerebro-cerebellar and limbic systems to acupuncture stimulation at ST 36 as evidenced by fMRI	359	17.95	NEUROIMAGE	2005	4.7	11	article	mechanism
A neuroanatomical basis for electroacupuncture to drive the vagal-adrenal axis	348	87.00	NATURE	2021	50.5	12	article	mechanism
Characterizing Acupuncture Stimuli Using Brain Imaging with fMRI - A Systematic Review and Meta-Analysis of the Literature	347	26.69	PLOS ONE	2012	2.9	13	review	mechanism
Safety of Acupuncture: Results of a Prospective Observational Study with 229,230 Patients and Introduction of a Medical Information and Consent Form	342	21.38	FORSCHENDE KOMPLEMENTARMEDIZIN	2009	1.3 (2018)	14	article	safety
Acupuncture in patients with chronic low back pain - A randomized controlled trial	326	17.16	ARCHIVES OF INTERNAL MEDICINE	2006	17.3 (2014)	15	article	treatment
Characteristic and incidental (placebo) effects in complex interventions such as acupuncture	303	15.15	BMJ-BRITISH MEDICAL JOURNAL	2005	93.7	16	article	effectiveness
Acupuncture for shoulder pain	302	15.10	COCHRANE DATABASE OF SYSTEMATIC REVIEWS	2005	8.8	17	review	treatment
A Randomized Trial Comparing Acupuncture, Simulated Acupuncture, and Usual Care for Chronic Low Back Pain	298	18.63	ARCHIVES OF INTERNAL MEDICINE	2009	17.3 (2014)	18	article	treatment
Meta-analysis: Acupuncture for low back pain	292	14.60	ANNALS OF INTERNAL MEDICINE	2005	19.6	19	review	treatment
Efficacy of acupuncture for the prophylaxis of migraine: a multicentre randomised controlled clinical trial	287	15.11	LANCET NEUROLOGY	2006	46.6	20	article	treatment

Effects of electroacupuncture versus manual acupuncture on the human brain as measured by fMRI	284	14.20	HUMAN BRAIN MAPPING	2005	3.5	21	article	mechanism
Acupuncture de qi, from qualitative history to quantitative measurement	275	15.28	JOURNAL OF ALTERNATIVE AND COMPLEMENTARY MEDICINE	2007	2.4	22	review	other
Acupuncture and knee osteoarthritis -: A three-armed randomized trial	275	14.47	ANNALS OF INTERNAL MEDICINE	2006	19.6	23	article	treatment
tension-type headache: randomised controlled trial	269	13.45	BMJ-BRITISH MEDICAL JOURNAL	2005	93.7	24	article	treatment
Expectancy and belief modulate the neuronal substrates of pain treated by acupuncture	267	13.35	NEUROIMAGE	2005	4.7	25	article	mechanism
Acupuncture for migraine prophylaxis	258	16.13	COCHRANE DATABASE OF SYSTEMATIC REVIEWS	2009	8.8	26	review	treatment
Effect of Electroacupuncture on Urinary Leakage Among Women With Stress Urinary Incontinence A Randomized Clinical Trial	251	31.38	JAMA-JOURNAL OF THE AMERICAN MEDICAL ASSOCIATION	2017	63.5	27	article	treatment
The Long-term Effect of Acupuncture for Migraine Prophylaxis A Randomized Clinical Trial	248	31.00	JAMA INTERNAL MEDICINE	2017	22.3	28	article	treatment
Acupuncture treatment for pain: systematic review of randomised clinical trials with acupuncture, placebo acupuncture, and no acupuncture groups	240	15.00	BMJ-BRITISH MEDICAL JOURNAL	2009	93.7	29	review	treatment

Acupuncture and dry-needling for low back pain: An updated systematic review within the framework of the Cochrane Collaboration	236	11.80	SPINE	2005	2.7	30	review	treatment
Clinical Evidence for Association of Acupuncture and Acupressure With Improved Cancer Pain A Systematic Review and Meta-Analysis	232	46.40	JAMA ONCOLOGY	2020	22.3	31	review	treatment
Acupuncture analgesia: A review of its mechanisms of actions	229	13.47	AMERICAN JOURNAL OF CHINESE MEDICINE	2008	4.8	32	review	mechanism
Acupuncture modulates resting state connectivity in default and sensorimotor brain networks	228	13.41	PAIN	2008	5.9	33	article	mechanism
Study of acupuncture for low back pain in recent 20 years: a bibliometric analysis via CiteSpace	225	28.13	JOURNAL OF PAIN RESEARCH	2017	2.5	34	review	treatment
Mechanisms of Acupuncture Therapy in Ischemic Stroke Rehabilitation: A Literature Review of Basic Studies	224	28.00	INTERNATIONAL JOURNAL OF MOLECULAR SCIENCES	2017	4.9	35	review	mechanism
Systematic Review of Acupuncture in Cancer Care: A Synthesis of the Evidence	220	18.33	JOURNAL OF CLINICAL ONCOLOGY	2013	42.1	36	review	treatment
Acupuncture analgesia: Areas of consensus and controversy	216	15.43	PAIN	2011	5.9	37	review	mechanism
Effect of Acupuncture vs Sham Acupuncture or Waitlist Control on Joint Pain Related to Aromatase Inhibitors Among Women With Early-Stage Breast Cancer A Randomized Clinical Trial	211	30.14	JAMA-JOURNAL OF THE AMERICAN MEDICAL ASSOCIATION	2018	63.5	38	article	treatment

Acupuncture and related techniques for postoperative pain: a systematic review of randomized controlled trials	211	12.41	BRITISH JOURNAL OF ANAESTHESIA	2008	9.1	39	review	treatment
Somatotopic Organization and Intensity Dependence in Driving Distinct NPY-Expressing Sympathetic Pathways by Electroacupuncture	209	41.80	NEURON	2020	14.7	40	article	mechanism
Traditional Chinese acupuncture and placebo (sham) acupuncture are differentiated by their effects on μ-opioid receptors (MORs)	206	12.88	NEUROIMAGE	2009	4.7	41	article	mechanism
The Salient Characteristics of the Central Effects of Acupuncture Needling: Limbic-Paralimbic-Neocortical Network Modulation	202	12.63	HUMAN BRAIN MAPPING	2009	3.5	42	article	mechanism
Randomized, Blinded, Sham-Controlled Trial of Acupuncture for the Management of Aromatase Inhibitor-Associated Joint Symptoms in Women With Early-Stage Breast Cancer	200	13.33	JOURNAL OF CLINICAL ONCOLOGY	2010	42.1	43	article	treatment
Acupuncture for Chronic Severe Functional Constipation A Randomized Trial	198	22.00	ANNALS OF INTERNAL MEDICINE	2016	19.6	44	article	treatment
Acupuncture - a critical analysis	197	10.37	JOURNAL OF INTERNAL MEDICINE	2006	9	45	review	other
Pragmatic randomized trial evaluating the clinical and economic effectiveness of acupuncture for chronic low back pain	196	10.32	AMERICAN JOURNAL OF EPIDEMIOLOGY	2006	5	46	article	treatment

Acupuncture for the prevention of episodic migraine	193	21.44	COCHRANE DATABASE OF SYSTEMATIC REVIEWS	2016	8.8	47	review	treatment
Acupuncture treatment for chronic knee pain: a systematic review	193	10.72	RHEUMATOLOGY	2007	4.7	48	review	treatment
Acupuncture for Chronic Knee Pain A Randomized Clinical Trial	192	17.45	JAMA-JOURNAL OF THE AMERICAN MEDICAL ASSOCIATION	2014	63.5	49	article	treatment
Acupuncture for peripheral joint osteoarthritis	191	12.73	COCHRANE DATABASE OF SYSTEMATIC REVIEWS	2010	8.8	50	review	treatment
Stimulation of the wrist acupuncture point PC6 for preventing postoperative nausea and vomiting	181	18.10	COCHRANE DATABASE OF SYSTEMATIC REVIEWS	2015	8.8	51	review	treatment
Acupuncture analgesia: I. The scientific basis	181	10.65	ANESTHESIA AND ANALGESIA	2008	4.6	52	review	mechanism
Acupuncture for Cancer-Related Fatigue in Patients With Breast Cancer: A Pragmatic Randomized Controlled Trial	180	13.85	JOURNAL OF CLINICAL ONCOLOGY	2012	42.1	53	article	treatment
Acupuncture for Chronic Low Back Pain.	176	11.73	NEW ENGLAND JOURNAL OF MEDICINE	2010	96.3	54	letter	treatment
Neuroimaging acupuncture effects in the human brain	175	9.72	JOURNAL OF ALTERNATIVE AND COMPLEMENTARY MEDICINE	2007	2.4	55	review	mechanism
The neuroimmune basis of anti-inflammatory acupuncture	174	9.67	INTEGRATIVE CANCER THERAPIES	2007	2.9	56	review	mechanism

Acupuncture for functional gastrointestinal disorders	174	9.16	JOURNAL OF GASTROENTEROLOGY	2006	6.9	57	article	mechanism
The Efficacy of Acupuncture in Post-Operative Pain Management: A Systematic Review and Meta-Analysis	170	18.89	PLOS ONE	2016	2.9	58	review	treatment
Electrical properties of acupuncture points and meridians: A systematic review	170	10.00	BIOELECTROMAGNETICS	2008	1.8	59	review	mechanism
Meta-analysis: Acupuncture for osteoarthritis of the knee	169	9.39	ANNALS OF INTERNAL MEDICINE	2007	19.6	60	review	treatment
The mechanism of acupuncture and clinical applications	167	8.79	INTERNATIONAL JOURNAL OF NEUROSCIENCE	2006	1.7	61	article	mechanism
Pretreatment With Electroacupuncture Induces Rapid Tolerance to Focal Cerebral Ischemia Through Regulation of Endocannabinoid System	166	10.38	STROKE	2009	7.9	62	article	mechanism
Acupuncture in Poststroke Rehabilitation A Systematic Review and Meta-Analysis of Randomized Trials	164	10.93	STROKE	2010	7.9	63	review	treatment
Acupuncture use in the United States: Findings from the National Health Interview Survey	164	8.63	JOURNAL OF ALTERNATIVE AND COMPLEMENTARY MEDICINE	2006	2.4	64	article	other
Randomized trial of acupuncture to lower blood pressure	159	8.83	CIRCULATION	2007	35.6	65	article	treatment
Acupuncture for depression	158	22.57	COCHRANE DATABASE OF SYSTEMATIC REVIEWS	2018	8.8	66	review	treatment

Acupuncture for Chronic Pain	156	14.18	JAMA-JOURNAL OF THE AMERICAN MEDICAL ASSOCIATION	2014	63.5	67	review	treatment
Hypothalamus and amygdala response to acupuncture stimuli in carpal tunnel syndrome	156	8.67	PAIN	2007	5.9	68	article	mechanism
Acupuncture Versus Venlafaxine for the Management of Vasomotor Symptoms in Patients With Hormone Receptor-Positive Breast Cancer: A Randomized Controlled Trial	155	10.33	JOURNAL OF CLINICAL ONCOLOGY	2010	42.1	69	article	treatment
A Randomized clinical trial of acupuncture compared with sham acupuncture in Fibromyalgia	155	7.75	ANNALS OF INTERNAL MEDICINE	2005	19.6	70	article	treatment
Acupuncture as Adjunctive Therapy for Chronic Stable Angina: A Randomized Clinical Trial	154	25.67	JAMA INTERNAL MEDICINE	2019	22.3	71	article	treatment
Imbalance between Pro- and Anti-inflammatory Cytokines, and between Th1 and Th2 Cytokines in Depressed Patients: The Effect of Electroacupuncture or Fluoxetine Treatment	153	9.56	PHARMACOPSYCHIATRY	2009	3.6	72	article	mechanism
Global trends and performances of acupuncture research	149	10.64	NEUROSCIENCE AND BIOBEHAVIORAL REVIEWS	2011	7.6	73	review	other
Subcutaneous tissue fibroblast cytoskeletal remodeling induced by acupuncture: Evidence for a mechanotransduction-based mechanism	149	7.84	JOURNAL OF CELLULAR PHYSIOLOGY	2006	4.5	74	article	mechanism

The effectiveness and safety of acupuncture therapy in depressive disorders: Systematic review and meta-analysis	147	9.80	JOURNAL OF AFFECTIVE DISORDERS	2010	4.9	75	review	treatment
The management of cancer-related fatigue after chemotherapy with acupuncture and acupressure: A randomised controlled trial	146	8.11	COMPLEMENTARY THERAPIES IN MEDICINE	2007	3.3	76	article	treatment
Inserting Needles Into the Body: A Meta-Analysis of Brain Activity Associated With Acupuncture Needle Stimulation	145	12.08	JOURNAL OF PAIN	2013	4	77	review	mechanism
How large are the nonspecific effects of acupuncture? A meta-analysis of randomized controlled trials	144	9.60	BMC MEDICINE	2010	7.1	78	review	effectiveness
Randomised controlled trial of a short course of traditional acupuncture compared with usual care for persistent non-specific low back pain	143	7.53	BMJ-BRITISH MEDICAL JOURNAL	2006	93.7	79	article	treatment
Acupuncture as an adjunct to exercise based physiotherapy for osteoarthritis of the knee: randomised controlled trial	142	7.89	BMJ-BRITISH MEDICAL JOURNAL	2007	93.7	80	article	treatment
Psychophysical outcomes from a randomized pilot study of manual, electro, and sham acupuncture treatment on experimentally induced thermal pain	142	7.10	JOURNAL OF PAIN	2005	4	81	article	other
Efficacy and safety of acupuncture treatment on primary insomnia: a randomized controlled trial	139	17.38	SLEEP MEDICINE	2017	3.8	82	article	treatment

Impact of electro-acupuncture and physical exercise on hyperandrogenism and oligo/amenorrhea in women with polycystic ovary syndrome: a randomized controlled trial	139	9.93	AMERICAN JOURNAL OF PHYSIOLOGY-ENDOCRINOLOGY AND METABOLISM	2011	4.2	83	article	treatment
History of Acupuncture Research	138	11.50	INTERNATIONAL REVIEW OF NEUROBIOLOGY	2013	4.2 (2021)	84	review	other
Acupuncture inhibits neuroinflammation and gut microbial dysbiosis in a mouse model of Parkinson's disease	137	27.40	BRAIN BEHAVIOR AND IMMUNITY	2020	8.8	85	article	mechanism
A Randomized Controlled Trial of Acupuncture for Osteoarthritis of the Knee: Effects of Patient-Provider Communication	137	9.13	ARTHRITIS CARE & RESEARCH	2010	3.7	86	article	treatment
Effect of Acupuncture and Clomiphene in Chinese Women With Polycystic Ovary Syndrome A Randomized Clinical Trial	136	17.00	JAMA-JOURNAL OF THE AMERICAN MEDICAL ASSOCIATION	2017	63.5	87	article	treatment
Acupuncture Modulates Resting State Hippocampal Functional Connectivity in Alzheimer Disease	136	12.36	PLOS ONE	2014	2.9	88	article	mechanism
Gastrointestinal motility disorders and acupuncture	135	9.00	AUTONOMIC NEUROSCIENCE-BASIC & CLINICAL	2010	3.2	89	review	treatment
Acupuncture and other physical treatments for the relief of pain due to osteoarthritis of the knee: network meta-analysis	134	11.17	OSTEOARTHRITIS AND CARTILAGE	2013	7.2	90	review	treatment
Acupuncture for treating fibromyalgia	133	11.08	COCHRANE DATABASE OF SYSTEMATIC REVIEWS	2013	8.8	91	review	treatment

Western medical acupuncture: a definition	132	8.25	ACUPUNCTURE IN MEDICINE	2009	2.4	92	article	other
Is acupuncture analgesia an expectancy effect? Preliminary evidence based on participants' perceived assignments in two placebo-controlled trials	132	6.60	EVALUATION & THE HEALTH PROFESSIONS	2005	2.2	93	article	treatment
Effects of acupuncture on rates of pregnancy and live birth among women undergoing in vitro	131	7.71	BMJ-BRITISH MEDICAL JOURNAL	2008	93.7	94	review	treatment
Low-frequency electroacupuncture and physical exercise decrease high muscle sympathetic nerve activity in polycystic ovary syndrome	129	8.06	AMERICAN JOURNAL OF PHYSIOLOGY-REGULATORY, INTEGRATIVE AND COMPARATIVE PHYSIOLOGY	2009	2.2	95	article	treatment
Acupoint-specific fMRI patterns in human brain	129	6.45	NEUROSCIENCE LETTERS	2005	2.5	96	article	mechanism
Cryogel/hydrogel biomaterials and acupuncture combined to promote diabetic skin wound healing	128	32.00	BIOMATERIALS	2021	12.8	97	article	treatment
Randomized, controlled trial of acupuncture for the treatment of hot flashes in breast cancer patients	128	7.11	JOURNAL OF CLINICAL ONCOLOGY	2007	42.1	98	article	treatment
Safety and efficacy of acupuncture in children - A review of the evidence	127	7.47	JOURNAL OF PEDIATRIC HEMATOLOGY ONCOLOGY	2008	0.9	99	review	safety
Acupuncture for posttraumatic stress disorder - A randomized controlled pilot trial	127	7.06	JOURNAL OF NERVOUS AND MENTAL DISEASE	2007	1.6	100	article	treatment
