# Supplementary material for: From tradition to evidence-based medicine: A two-decade bibliometric analysis of acupuncture efficacy, neural mechanisms, and therapeutic integration (2005–2024)
Source: Medicine (Baltimore). 2026 Nov 6;105(45):e51353. doi: 10.1097/MD.0000000000051353 (PMC13747620; doi:10.1097/MD.0000000000051353)
Supplement: Supplementary file 2 [file medi-105-e51353-s002.pdf]

id	country	documents	citations	total link strength
17	usa	51	12011	51
11	peoples r china	29	6177	35
5	germany	23	6800	37
4	england	21	4819	25
14	switzerland	7	2747	9
12	south korea	6	1213	14
15	taiwan	6	1110	2
1	australia	5	1024	7
2	canada	5	983	9
9	netherlands	4	728	11
13	sweden	3	407	4
6	italy	2	452	7
10	norway	2	302	3
3	denmark	1	243	0
7	japan	1	237	3
8	mexico	1	452	1
16	turkey	1	168	0
