# Supplementary material for: From tradition to evidence-based medicine: A two-decade bibliometric analysis of acupuncture efficacy, neural mechanisms, and therapeutic integration (2005–2024)
Source: Medicine (Baltimore). 2026 Nov 6;105(45):e51353. doi: 10.1097/MD.0000000000051353 (PMC13747620; doi:10.1097/MD.0000000000051353)
Supplement: Supplementary file 3 [file medi-105-e51353-s003.pdf]

id	organization	documents	citations	total link strength
163	tech univ munich	14	4440	40
185	univ maryland	13	3285	38
98	massachusetts gen hosp	12	2713	27
22	charite univ med ctr	8	2787	21
64	harvard univ	8	1841	20
103	mem sloan kettering canc ctr	7	2182	22
25	china acad chinese med sci	5	1238	22
11	beijing univ chinese med	5	944	20
92	kyung hee univ	5	1054	13
93	logan coll chiropract	5	1192	12
214	univ zurich hosp	5	1921	12
21	charite	4	1283	21
212	univ york	4	1535	20
154	sichuan univ	4	862	19
210	univ vermont	4	688	12
23	chengdu univ tradit chinese med	4	894	11
189	univ munich	4	894	8
26	china med univ	4	779	4
152	shanghai univ tradit chinese med	3	598	17
201	univ southampton	3	1389	14
195	univ plymouth	3	588	7
177	univ keele	3	526	6
52	fudan univ	3	1342	5
138	peninsula med sch	3	679	5
137	peking univ	3	519	4
144	ruhr univ bochum	3	1034	0
41	daemen coll	2	456	15
74	hubei prov hosp tradit chinese med	2	456	15
81	jiangsu prov hosp tradit chinese med	2	456	15
166	tianjin univ tradit chinese med	2	456	15
86	keele univ	2	1210	12
17	capital med univ	2	337	10
133	oregon coll oriental med	2	361	10
53	georgetown univ	2	322	9
187	univ michigan	2	397	9
55	grp hlth cooperat puget sound	2	346	8
213	univ zurich	2	826	8
42	dana farber canc inst	2	1090	7
215	vrije univ amsterdam	2	360	6
108	minist educ	2	286	5
6	asia univ	2	378	4
7	baylor coll med	2	353	4
76	hunan univ tradit chinese med	2	406	4
87	kings coll london	2	320	3
172	univ exeter	2	391	3
30	chinese peoples armed police forces	2	266	2
140	rmit univ	2	368	2
176	univ hong kong	2	289	2
183	univ manchester	2	325	2
79	inst work & hlth	2	526	1
94	london sch hyg & trop med	2	359	1
112	monash univ	2	497	1
203	univ texas md anderson canc ctr	2	358	1
36	columbia univ	2	414	0
43	duke univ	2	385	0
85	karolinska inst	2	270	0
