# Supplementary material for: From tradition to evidence-based medicine: A two-decade bibliometric analysis of acupuncture efficacy, neural mechanisms, and therapeutic integration (2005–2024)
Source: Medicine (Baltimore). 2026 Nov 6;105(45):e51353. doi: 10.1097/MD.0000000000051353 (PMC13747620; doi:10.1097/MD.0000000000051353)
Supplement: Supplementary file 4 [file medi-105-e51353-s004.pdf]

id	author	documents	citations	total link strength
273	linde, klaus	14	4440	76
498	witt, claudia m.	10	3867	58
33	brinkhaus, benno	9	2912	38
26	berman, brian m.	9	2155	20
338	napadow, vitaly	8	2068	27
495	willich, sn	7	2460	48
238	lao, lixing	7	1599	21
297	macpherson, hugh	5	1678	21
323	melchart, d	4	1503	37
414	streng, a	4	1503	37
459	wagenpfeil, s	4	1503	37
403	sherman, karen j.	4	1700	21
306	manheimer, eric	4	746	16
457	vickers, andrew j.	4	1946	14
266	liang, fanrong	3	753	16
116	foster, nadine e.	3	1353	13
264	li, ying	3	634	12
29	bouter, lex m.	3	491	11
225	kong, jian	3	622	2
270	lin, jaung-geng	3	630	0
174	hoppe, a	2	724	20
189	hummelsberger, j	2	722	20
366	pfaffenrath, v	2	724	20
484	weidenhammer, w	2	724	20
191	irnich, d	2	597	19
200	jena, s	2	779	17
464	walther, hu	2	779	17
254	lewith, george	2	1210	13
45	chang, xiaorong	2	406	12
421	sun, xin	2	406	12
455	vertosick, emily a.	2	681	12
509	wu, xi	2	406	12
551	zhao, ling	2	406	12
554	zheng, hui	2	406	12
156	haramati, aviad	2	322	11
5	allais, gianni	2	452	10
149	hammerschlag, richard	2	361	10
235	langevin, helene m.	2	361	10
482	wayne, peter m.	2	361	10
490	white, adrian r.	2	452	10
159	harris, richard e.	2	397	9
188	hui, kks	2	645	8
230	kwong, kk	2	645	8
277	liu, j	2	645	8
304	makris, n	2	645	8
352	pach, daniel	2	689	8
504	wruck, katja	2	539	8
357	park, kyungmo	2	576	7
40	capodice, jillian l.	2	414	6
74	crew, katherine d.	2	414	6
142	greenlee, heather	2	414	6
168	hershman, dawn l.	2	414	6
88	dhond, rupali p.	2	404	5
124	furlan, ad	2	526	5
216	kettner, norman	2	404	5
443	tsukayama, h	2	526	5
198	janson, per olof	2	270	4

199	jedel, elizabeth	2	270	4
411	stener-victorin, elisabet	2	270	4
281	liu, shenbin	2	580	2
293	ma, qiufu	2	580	2
100	ernst, e	2	490	1
355	park, hi-joon	2	288	1
153	han, ji-sheng	2	366	0
302	maier, christoph	2	747	0
332	molassiotis, alexander	2	325	0
