# Supplementary material for: From tradition to evidence-based medicine: A two-decade bibliometric analysis of acupuncture efficacy, neural mechanisms, and therapeutic integration (2005–2024)
Source: Medicine (Baltimore). 2026 Nov 6;105(45):e51353. doi: 10.1097/MD.0000000000051353 (PMC13747620; doi:10.1097/MD.0000000000051353)
Supplement: Supplementary file 5 [file medi-105-e51353-s005.pdf]

Title	TC	ACY	Journal	First Author	Year	IF	Number	Type	CorrespondingAuther
Evidence on acupuncture therapies is underused in clinical practice and health policy	104	34.67	BMJ-BRITISH MEDICAL JOURNAL	Liming Lu	2022	93.7	1	Editorial Material	Nenggui Xu
Anti-Inflammatory Effects of Acupuncture at ST36 Point: A Literature Review in Animal Studies	99	33.00	FRONTIERS IN IMMUNOLOGY	Ji-Eun Oh	2022	5.7	2	review	Seung-Nam Kim
Understandings of acupuncture application and mechanisms	89	29.67	AMERICAN JOURNAL OF TRANSLATIONAL RESEARCH	Lin, Jaung-Geng	2022	1.7	3	review	Lin, Jaung-Geng
Effect of Electroacupuncture on Insomnia in Patients With Depression A Randomized Clinical Trial	76	25.33	JAMA NETWORK OPEN	Yin, X	2022	10.5	4	article	Xu, Shifen
Potential Mechanisms and Clinical Effectiveness of Acupuncture in Depression	74	24.67	CURRENT NEUROPHARMACOLOGY	Yang, NN	2022	4.8	5	review	Liu, Cun-Zhi
The immunomodulatory mechanisms for acupuncture practice	67	33.50	FRONTIERS IN IMMUNOLOGY	Wang, M	2023	5.7	6	review	Liu, Shenbin
Methodological challenges in design and conduct of randomised controlled trials in acupuncture	67	22.33	BMJ-BRITISH MEDICAL JOURNAL	Fei, YT	2022	93.7	7	Editorial Material	Liu, Jian-Ping
Effectiveness of Acupuncture for Anxiety Among Patients With Parkinson Disease A Randomized Clinical Trial	61	20.33	JAMA NETWORK OPEN	Fan, JQ	2022	10.5	8	article	Zhuang, Li-Xing
Electroacupuncture Alleviates LPS-Induced ARDS Through α7 Nicotinic Acetylcholine Receptor-Mediated Inhibition of Ferroptosis	53	17.67	FRONTIERS IN IMMUNOLOGY	Zhang, YG	2022	5.7	9	article	Yang, Hao，Lv, Xin

Electroacupuncture vs Sham Electroacupuncture in the Treatment of Postoperative Ileus After Laparoscopic Surgery for Colorectal Cancer A Multicenter, Randomized Clinical Trial	50	25.00	JAMA SURGERY	Wang, Y	2023	15.9	10	review	Liu, Cun-Zhi
